# Thymosin beta-4 improves endothelial function and reparative potency of diabetic endothelial cells differentiated from patient induced pluripotent stem cells

**DOI:** 10.1186/s13287-021-02687-x

**Published:** 2022-01-10

**Authors:** Liping Su, Xiaocen Kong, Szejie Loo, Yu Gao, Bingli Liu, Xiaofei Su, Rinkoo Dalan, Jianhua Ma, Lei Ye

**Affiliations:** 1 National Heart Research Institute Singapore, National Heart Centre Singapore, Singapore, 169609 Singapore; 2 Department of Endocrinology, Nanjing First Hospital, Nanjing Medical University, Nanjing, 210029 China; 3 Department of Cardiology, Ren Ji Hospital, School of Medicine, Shanghai Jiao Tong University, Shanghai, 200127 China; 4 Department of Endocrinology, Tan Tock Seng Hospital, Lee Kong Chian School of Medicine, Nanyang Technological University, Singapore, Singapore

**Keywords:** Endothelium, Endothelial function, Senescence, Mitochondrial function, Thymosin

## Abstract

**Background:**

Prior studies show that signature phenotypes of diabetic human induced pluripotent stem cells derived endothelial cells (dia-hiPSC-ECs) are disrupted glycine homeostasis, increased senescence, impaired mitochondrial function and angiogenic potential as compared with healthy hiPSC-ECs. In the current study, we aimed to assess the role of thymosin β-4 (Tb-4) on endothelial function using dia-hiPSC-ECs as disease model of endothelial dysfunction.

**Methods and results:**

Using dia-hiPSC-ECs as models of endothelial dysfunction, we determined the effect of Tb-4 on cell proliferation, senescence, cyto-protection, protein expression of intercellular adhesion molecule-1 (ICAM-1), secretion of endothelin-1 and MMP-1, mitochondrial membrane potential, and cyto-protection in vitro and angiogenic potential for treatment of ischemic limb disease in a mouse model of type 2 diabetes mellitus (T2DM) in vivo. We found that 600 ng/mL Tb4 significantly up-regulated AKT activity and Bcl-XL protein expression, enhanced dia-hiPSC-EC viability and proliferation, limited senescence, reduced endothelin-1 and MMP-1 secretion, and improved reparative potency of dia-hiPSC-ECs for treatment of ischemic limb disease in mice with T2DM. However, Tb4 had no effect on improving mitochondrial membrane potential and glycine homeostasis and reducing intercellular adhesion molecule-1 protein expression in dia-hiPSC-ECs.

**Conclusions:**

Tb-4 improves endothelial dysfunction through enhancing hiPSC-EC viability, reducing senescence and endothelin-1 production, and improves angiogenic potency in diabetes.

## Introduction

Vascular endothelial cells (ECs) play an important role in maintaining cardiovascular homeostasis through mediating vascular tone, cell adhesion, the homeostasis of clotting, and fibrinolysis [1, 2]. However, hyperglycemia causes endothelial activation, dysfunction, and senescence, all of which constitute molecule and cellular basis for both microvascular and macrovascular complications in diabetes [3–11]. Previously, we established human induced pluripotent stem cells (hiPSCs) derived from patients with type 2 diabetes mellitus (T2DM) and differentiated them into endothelial cells (dia-hiPSC-ECs) [12]. We identified signature phenotypes in dia-hiPSC-ECs: disrupted glycine homeostasis, reduced cell proliferation, increased senescence, and impaired mitochondrial function and angiogenesis. Treatments to improve endothelial function, cell viability, and angiogenesis and correct glycine metabolism may be promising therapeutic targets for preventing microvascular and macrovascular complications in diabetes.

Thymosin β4, a 5 kDa polypeptide, is composed of 43 amino acids [13]. It is the most abundant member of the β-thymosin family in mammalian tissue and is regarded as the main G-actin sequestering peptide [13]. It is known that Tb4 promotes wound-healing and tissue-repair. It promotes dermal healing [14] and corneal wound healing [15] and improves heart function after myocardial infarction[16–19]. Tb4 also promotes neuron survival and neurite outgrowth of cultured spinal cord neurons [20]. In addition, Tb4 also possesses angiogenic activity to promote EC migration, tubule formation, and angiogenesis [21]. Tb4 also promotes epicardial progenitor cells differentiation into ECs, thereby serving as a source of vascular progenitors for coronary vasculogenesis and angiogenesis [22].

Currently, there is little information on the role of Tb4 on diabetic ECs. Previously, we showed that Tb4 improved migration of CD34^+^/KDR^+^ circulating endothelial progenitor cells (EPCs) from diabetic fatty rats [23, 24]. Furthermore, in the current study, we aimed to use diabetic-hiPSC-ECs as diabetic EC models to study the role of Tb4 on proliferation, viability, senescence, and angiogenesis of diabetic ECs.

## Methods

### Culture and differentiation of hiPSC

Two diabetic hiPSC lines, DP2C8iPS and DP3C6iPS cells, were described previously[12, 25]. Both cell lines were reprogrammed from dermal fibroblasts of two adult patients with T2DM. hiPSCs were cultured in a feeder-free system with a 1:2 mixture of E8/mTeSR (STEMCELL Technologies, Canada) and were passaged every 4–5 days with ReleSR (STEMCELL Technologies). The characterization of both cell lines were described previously [12].

The EC differentiation protocol has been described previously by us [25, 26]. The differentiated hiPSC-ECs positive for CD31 expression and for both CD31 and CD144 expression were collected by fluorescence activated cell sorting (FACS) and expanded. hiPSC-ECs were cultured in EGM2-MV medium (Lonza, Switzerland) supplemented with B27, vascular endothelial growth factor-165 (VEGF), and SB-431542 (SB) [25, 26].

### Biological function of Tb4 on diabetic hiPSC-ECs in vitro

#### Cell population doubling time of diabetic hiPSC-ECs

Endothelial cell population doubling time was calculated within 7 days post-sorting. Briefly, ECs were cultured in 6-well plates in EGM which was daily supplemented with or without Tb4 protein (Prospec, USA). The medium was changed every 2 days. hiPSC-ECs were harvested and counted on day 7.

#### Tube formation

Tube formation were evaluated as described previously [25, 26]. Briefly, Tb4 treated or non-treated 2 × 10^4^ cells/well were seeded in 48-well plate that had been coated with Matrigel (Corning, USA) and incubated at 37 °C for 24 h. Numbers of node, junction, and branches and total branches length per magnification (4 ×) were quantified using angiogenesis analyzer of Image J.

#### Hypoxia treatment and lactate dehydrogenase (LDH) assay and DNA damage measurement

For assessment of cyto-protection by Tb4 on diabetic hiPSC-ECs, 5 × 10^4^ hiPSC-ECs/well were cultured in 24-well plate. After washing thrice with Dulbecco's phosphate-buffered saline (DPBS), hiPSC-ECs were cultured in 400 μL endothelial basal medium (EBM, Lonza) supplemented with or without Tb4 protein and cultured in an incubator with hypoxic condition for 24 h: 5% CO_2_, 94% N_2_, and 1% O_2_ [18].

The supernatant was collected to determine the intensity of LDH fluorescence in the supernatant using the Cytotoxicity detection kit (Roche, USA) per manufacturer’s instructions [27]. A human DNA fragmentation factor subunit beta ELISA kit (Abbexa, USA) was used to determine damaged DNA released into the supernatant according to manufacturer’s instructions [27].

To assess whether the cyto-protective effect of Tb4 is mediated by AKT and Bcl-XL, an AKT inhibitor, MK-2206 dihydrochloride (MedChemExpress, USA), and a Bcl-XL inhibitor, A-1155463 (MedChemExpress, USA) at 1 μM each were used. Inhibitors were added 30 min before 600 ng/mL Tb4 was added into cell culture medium. The supernatant was collected at 24 h after hypoxia treatment to determine the intensity of LDH fluorescence.

#### Endothelin-1 and MMP-1 secreted by dia-hiPSC-ECs

To determine secreted endothelin-1, a vasoconstrictor, and MMP-1, a marker of the senescence associated secretory phenotype, supernatant of hiPSC-ECs was collected for Western Blot analysis as described [12]. Mouse anti-endothelin-1 (Santa Cruz Biotech., USA) and rabbit anti-MMP-1 (Invitrogen, USA) at 1:250 and 1:200 dilutions, respectively, were used as primary antibody. Goat anti-mouse IgG (Perkin Elmer, USA) or rabbit IgG (Cell Signaling, USA) conjugated with horseradish peroxidase (HRP) at 1:1000 was used as secondary antibody. Endothelin-1 or MMP-1 protein expression was presented as fold change after comparing with that of DP2-EC or DP3-EC which was considered as 100%.

#### Adhesion molecule expressed by dia-hiPSC-ECs

Protein expression of ICAM-1 was determined using Western Blot as described [12]. Rabbit anti-ICAM-1 (1: 1000, Cell Signaling, USA) was used as primary antibody and goat anti-rabbit IgG conjugated with HRP (1: 4000, Cell Signaling, USA) was used as secondary antibody. The protein expression level of ICAM was normalized by GAPDH and expressed as percentage of GAPDH.

#### Senescence of ECs

To determine cell senescence, ECs were stained for β-galactosidase (β-gal) expression on day 14 post-sorting using a Senescence β-galactosidase staining kit (Cell Signaling, USA) as described [12]. Briefly, the total β-gal intensity in each picture was calculated using Image J and presented as β-gal intensity/cell = total β-gal intensity/cell number in each picture. EC lysates were used to assess the protein expression of p21 and p53 using Western Blot as described [12]. Mouse anti-p21, or aniti-p53, (both from Santa Cruz Biotech., USA), or rabbit anti-acetylated p53 (K382) (Ace-p53, R&D Systems, USA) primary antibody at 1:200 or 1:1000 dilution was used. Goat anti-mouse or rabbit IgG conjugated with HRP (Perkin Elmer, USA) at 1:1000 (p21), or 1:4000 (p53), or 1:2000 (Ace-p53) was used as secondary antibody. The protein expression level was normalized by GAPDH and expressed as percentage of GAPDH.

#### Mitochondrial membrane potential of dia-hiPSC-ECs

To determine mitochondrial membrane potential, after overnight cultured with EGM supplemented with DAPI, ECs on day-7 post-sorting were cultured with 1: 1 ratio of fresh EGM and JC-1 dye solution (Mitochondria staining kit, Sigma Aldrich, USA) for 30 min in an incubator at 37 °C. Then, cells were washed with DPBS and cultured in fresh EGM. Images of red fluorescence were randomly taken at 50 milli-seconds (ms) at 20× magnification using Olympus IX73 microscope and Cell Sens Standard software (Olympus, Japan). The fluorescence intensity of each cell was calculated as the overall fluorescence intensity divided by EC number in each image using Image J [12]. In addition, mSHMT protein levels in DP2-ECs and PD3-ECs were determined using Western Blot as described [12]. Mouse anti-mSHMT (from Santa Cruz Biotech., USA) at 1:500 dilution was used as primary antibody. Goat anti-mouse IgG conjugated with HRP (Perkin Elmer, USA) at 1: 4000 was used as secondary antibody. The protein expression level was normalized by GAPDH and expressed as percentage of GAPDH.

#### Western blot analysis

Total protein was isolated using PhosphoSafe™ Extraction Reagent (Merck, Germany) and protein concentration was determined using Bradford reagent (Bio-Rad Laboratories, USA) per manufacturer’s instruction [18, 28]. Western Blot was described previously [28–30]. Briefly, proteins were separated on SDS–polyacrylamide gel and were transferred onto nitrocellulose membrane. After blocking with 5% non-fat milk in Tris-buffered saline Tween-20 buffer (25 mM Tris, pH 7.5, 150 mM NaCl, and 0.1% Tween-20), the blots were incubated with primary antibodies: rabbit anti-glyceraldehyde phosphate dehydrogenase (GAPDH) at 1: 5000 dilution; p-AKT (S473) at 1: 2000 dilution; AKT at 1: 2000 dilution; Bcl-XL at 1: 1000 dilution (all from Cell Signaling, USA). Anti-rabbit IgG conjugated with HRP (1: 5000 for GAPDH and 1: 4000 for the rest) was used to detect the binding of antibodies. The binding of the specific antibody was detected using the SuperSignal Chemiluminescent Substrate kit (Pierce, USA) and visualized using ChemiDoc™ XRS + System (Bio-Rad, USA). The protein expression level was normalized by GAPDH and expressed as percentage of GAPDH.

### Biological function of Tb4 on diabetic hiPSC-ECs in vivo

#### Gelatin microsphere manufacturing

Gelatin microspheres were manufactured as described with modifications [18, 19]. Briefly, 5 mL of 10% gelatin (type A, Sigma-Aldrich, USA) solution at 50 °C was added into 45 °C olive, stirred, and cooled to 5 °C. 25 min later, chilled (4 °C) acetone was added to the olive oil to induce microsphere formation. Then, microspheres were collected, washed 5 times to remove the olive oil, air-dried at 4 °C, and resuspended in chilled (4 °C) 70% ethanol containing 1% glutaraldehyde (Sigma-Aldrich, USA) to induce cross-linking for 30 min at 4 °C. The mixture was neutralized with an equal volume of 0.1 M glycine (Sigma-Aldrich, USA). Cross-linked microspheres were collected by washing with ethanol solution and air-dried. Tb4 (Prospec, USA) was loaded into the microspheres by mixing 5 mg microspheres with 5 µL distilled H_2_O containing 5 µg Tb4.

#### Mouse model of hind-limb ischemia (HLI) and treatment

The animal experimental protocol and procedures were approved by the Institutional Animal Care and Use Committee of Singapore Health Services Pte Ltd. The animal model was developed as previously described [12, 25]. Briefly, 12-week-old KK.Cg-A^y^ mice (KK mice, Stock No: 002468, Jackson Lab, USA) with diabetes, which was confirmed by glucose tolerance test (GTT), were included. After overnight fast (about 16 h), mouse will be intra-peritoneally injected with 1 g/kg body weight of glucose diluted in saline (100 mg/mL) [29]. Blood samples from the tail vein were collected at 0 (before glucose injection), 30, 60, and 120 min after glucose injection. Blood glucose concentration were measured using Johnson and Johnson One Touch Ultra 2 Glucose Meter and Test Strips (USA).

Mice were anesthetized with 1.5–2% isoflurane. Their right hind limbs were shaved and the femoral arteries of the right limbs were ligated with 6-0 polypropylene sutures [12, 25]. Animals were randomly assigned to treatment with 1.2 × 10^6^ DP2-EC or DP3-EC (the DP2-EC or DP3-EC group), or with Tb4 treated 1.2 × 10^6^ DP2-EC or DP3-EC in 0.1 mL EBMs + 5 mg gelatin microspheres loaded with 5 μg Tb4 protein in 0.1 mL EBM (the DP2-EC + Tb4 or DP3-EC + Tb4 group), or only 0.1 mL EBM (the control group), or 5 mg gelatin microspheres loaded with 5 μg Tb4 protein in 0.1 mL EBM (the Tb4 group). Each animal group had 6 KK mice. Dia-hiPSC-ECs were cultured with (DP2-EC + Tb4 and DP3-EC + Tb4 Groups) or without (the DP2-EC and DP3-EC Groups) 600 ng/mL Tb4 for 5 days and were transplanted on day 7 post-sorting. The hiPSC-ECs, or gelatin microspheres, or basal medium were administered three days after HLI induction via 4 intramuscular injections into the center of the ligated area and the surrounding region along the femoral artery.

#### Laser doppler imaging

Mice were anesthetized with 1.5–2% isoflurane and their hind limbs were shaved. A PeriScan PIM 3 (Perimed, Sweden), a laser Doppler imaging system, was used to visualize limb perfusion as described [12, 25]. Measurements in the ischemic (right) limb were normalized to measurements in the left (non-ischemic) limb and expressed as a percentage.

#### Immunohistochemistry

To identify transplanted hiPSC-ECs, a primary antibody specifically against human CD31 (hCD31, mouse anti-human CD31-Biotin) was used and visualized by mouse anti-Biotin-VioBright 515 (both from Miltenyi Biotec, Germany) [12]. Fluorescence images were taken with an Olympus IX71 fluorescence microscope.

Neovascularization in ischemic limb was determined as described [31]. Cryosections were stained for CD31 expression (rabbit anti-CD31, Abcam, USA), which targets both human and mouse ECs), to evaluate total vessel density, and smooth muscle actin (SMA) expression (Cy3-conjugated mouse anti-SMA antibodies, Sigma-Aldrich), which targets smooth muscle cells (SMCs), to evaluate arteriole density. Vascular structures that were positive for CD31 expression and for both CD31 and SMA expression were counted for all animals in each group.

### Statistics

Data are presented as mean ± standard deviation (SD). Comparisons among groups were analyzed for significance via one-way analysis of variance (ANOVA) with the Tukey correction. Comparison between the two groups was performed with independent *T*-test. Analyses were performed with SPSS software. A value of *p* < 0.05 was considered significant.

## Results

### Tb4 (600 ng/mL) activates AKT activity and up-regulates Bcl-XL in dia-hiPSC-ECs

**Fig. 1:**
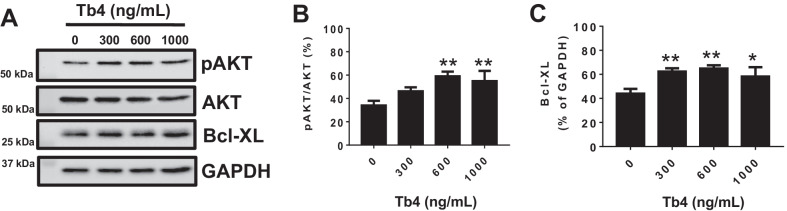
Dose dependent effect of Tb4 on AKT activity and Bcl-XL protein expression in dia-hiPSC-ECs. **A** Representative images of Western Blot analysis for protein expression of phosphorylated AKT (pAKT), AKT, and Bcl-XL in DP3-ECs as a function of Tb4 dosage. Quantification of pAKT/AKT (**B**) and Bcl-XL (**C**) in DP3-ECs (*n* = 3). Values are presented as means ± SD. One-way ANOVA (*p < 0.05; **p < 0.01, vs 0 ng/mL Tb4)

It is known that Tb4 is able to activate AKT to enhance cell viability. Thus, we first determined a dose dependent effect of Tb4 on AKT activation in dia-hiPSC-ECs under normoxia. Dia-hiPSC-ECs were cultured with (300, 600, and 1000 ng/mL Tb4) or without Tb4. Western blot showed that only 600 and 1000 ng/mL Tb4 significantly increased AKT activities (Fig. 1A, B). Furthermore, only 300 and 600 ng/mL Tb4 significantly up-regulated Bcl-XL protein expression (Fig. 1A, C). Thus, we used 600 ng/mL Tb4 in the rest experiments, except for hypoxic experiment.

### Tb4 (600 ng/mL) enhances dia-hiPSC-ECs potent for in vitro angiogenesis

**Fig. 2.**
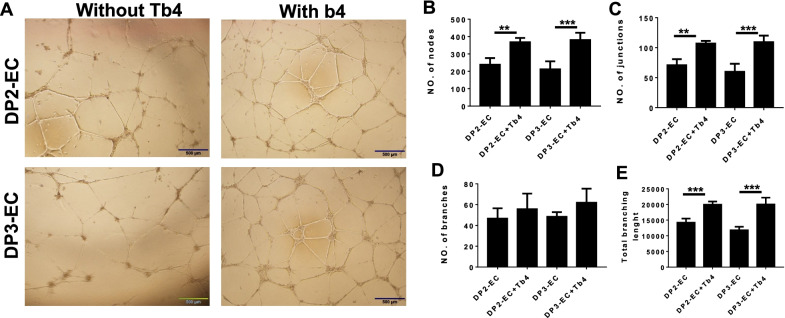
600 ng/mL Tb4 enhances angiogenic capapcity of dia-hiPSC-EC in vitro. **A** Representative images of tube formation of Tb4 treated and non-treated DP2-ECs or DP3-ECs on Matrigel. Quantification of numbers of nodes (**B**), junctions (**C**), and branches (**D**) and total branches length (**E**) formed by ECs on Matrigel. (Bar = 500 μm, *n* = 4). Values are presented as means ± SD. Independent *T*-test (**p < 0.01; ***p < 0.001)

To determine whether Tb4 could enhance angiogenic potent, Tb4 treated or non-treated DP2-ECs and DP3-ECs were cultured on Matrigel for tube formation assay (Fig. 2A). The formation of tubular structures was more extensive for Tb4 treated DP2-ECs and DP3-ECs than non-treated cells. The numbers of nodes and junctions and the total branching length were significantly higher in Tb4 treated DP2-ECs and DP3-ECs than Tb4 non-treated cells (Fig. 2B, C, E). Although the number of branches in Tb4 treated DP2-ECs and DP3-ECs had trends to be higher than non-treated ECs, no significant difference was found. (Fig. 2D). These results suggest that Tb4 enhanced dia-hiPSC-ECs angiogenic potent in vitro.

### Tb4 (600 ng/mL) increases dia-hiPSC-EC proliferation and reduces senescence

**Fig. 3.**
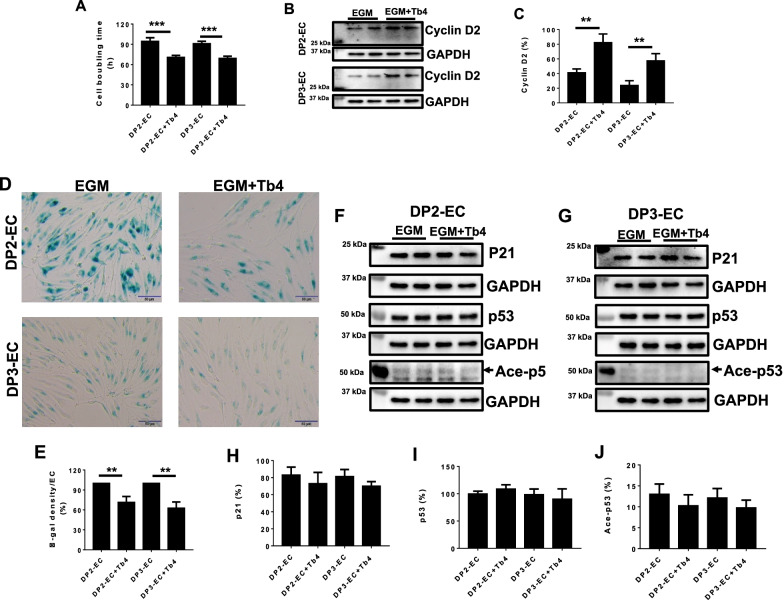
600 ng/mL Tb4 increases proliferation and reduces senescence in dia-hiPSC-ECs. **A** dia-hiPSC-EC population doubling time. **B** Representative images of Western Blot analysis for protein expression of Cyclin D2 in dia-hiPSC-ECs. **C** Quantification of cyclin D2 protein expression. **D** Representative images of β-gal staining (green color) in dia-hiPSC-ECs. **E** Quantification of β-gal density in dia-hiPSC-ECs. Representative images of Western Blot for p21, p53, and acetylated P53 (Ace-P53) protein expressions in DP2-ECs (**F**) and DP3-ECs (**G**). Quantification of p21 (**H**), p53 (**I**), and Ace-P53 (**J**) protein expression in DP2-ECs and DP3-ECs. (Bar = 50 μm, *n* = 4). Values are presented as means ± SD. Independent *T*-test (**p < 0.01; ***p < 0.001)

To determine the effect of Tb4 on cell proliferation, dia-hiPSC-ECs were cultured with 600 ng/mL Tb4. DP2-EC and DP3-EC doubling times were significantly reduced after cultured in 600 ng/mL Tb4 (Fig. 3A). Consistent with this, Western Blot showed that cyclin D2 protein expression significantly increased in DP2-ECs and DP3-ECs after cultured with 600 ng/mL Tb4 (Fig. 3B, C).

A cell senescence assay showed that β-gal protein expression significantly decreased in DP2-ECs and DP3-ECs after cultured with 600 ng/mL Tb4 (Fig. 3D, E). Western Blot showed that p21, P53, and Ace-P53 protein expression did not change significantly in DP2-ECs and DP3-ECs after cultured with 600 ng/mL Tb4 (Fig. 3F–J). These results suggest that change of p21 and p53 did not contribute to increased dia-hiPSC-EC proliferation and decreased senescence.

### Tb4 (600 ng/mL) reduces endothelin-1 and MMP-1 secretion

**Fig. 4.**
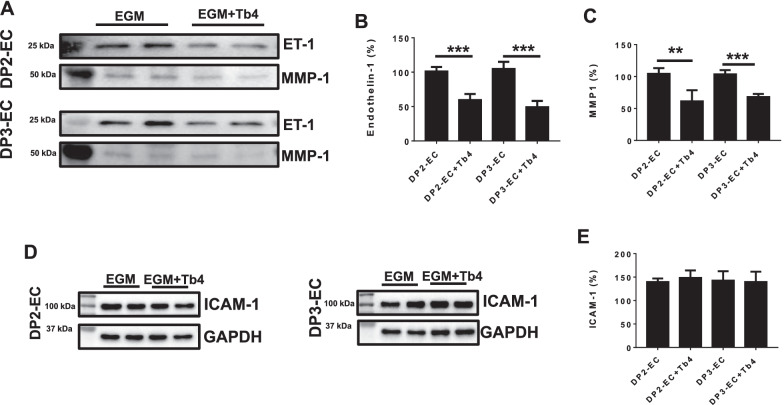
600 ng/mL Tb4 decreases endothelin-1 and MMP-1 secretion and does not reduce ICAM-1 expression. **A** Representative images of Western Blot analysis for protein expression of endothelin-1 (ET-1) and MMP-1 in supernatant of DP2-ECs and DP3-ECs. Quantification of endothelin-1 (**B**) and MMP-1 (**C**) protein expression. **D** Representative images of Western Blot analysis for protein expression of ICAM-1 in dia-iPSC-ECs. **E** Quantification of ICAM-1 protein expression (*n* = 4). Values are presented as means ± SD. Independent *T*-test (**p < 0.01; ***p < 0.001)

Both endothelin-1 and MMP-1 in supernatant were analyzed using Western Blot (Fig. 4A–C). Endothelin-1 protein in supernatant significantly reduced in DP2-EC and DP3-EC after treated with 600 ng/mL Tb4. MMP-1, a marker that makes up the senescence associated secretory phenotype (SASP) [32–34], also reduced significantly in the supernatants of DP2-ECs and DP3-ECs after treated with 600 ng/mL. As up-regulated MMP-1 has been seen in senescent cells and belongs to the SASP, its reduction indicates that DP2-ECs and DP3-ECs have better viability after treated with Tb4.

### Tb4 (600 ng/mL) does not reduce ICAM-1 protein expression and improve mSHMT protein expression and mitochondrial membrane potential in dia-hiPSC-ECs

**Fig. 5.**
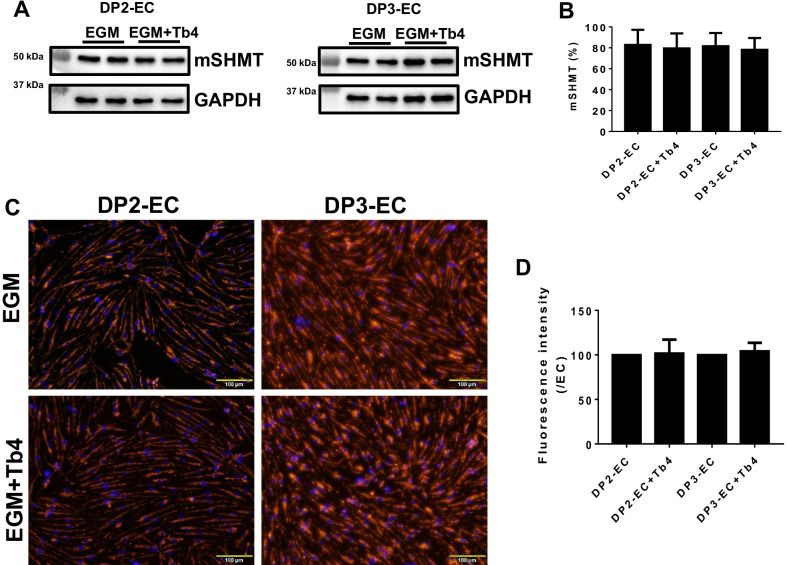
600 ng/mL Tb4 does not increase mSHMT protein expression and improve mitochondrial membrane potential. Representative images of Western Blot analysis for protein expression of mSHMT in DP2-ECs and DP3-ECs (**A**). **B** Quantification of mSHMT protein expression. **C** JC-1 staining to visualize mitochondrial membrane potential. **D** Quantification of hiPSC-EC mitochondrial membrane potential (*n* = 4). Values are presented as means ± SD. Independent *T*-test

We found that ICAM-1 protein expression significantly up-regulated and mitochondrial membrane potential significantly reduced in DP2-ECs and DP3-ECs in a previous study [12]. Thus, we measured ICAM-1 protein expression and mitochondrial membrane potential in dia-hiPSC-ECs after treated with Tb4. Western blot showed that ICAM-1 protein expression level was unchanged after treated with 600 ng/mL Tb4 (Fig. 4D, E). Similarly, protein expression level of mitochondrial serine hydroxymethyltransferase (mSHMT) were unchanged in DP2-EC and DP3-ECs after treated with 600 ng/mL Tb4 (Fig. 5A, B). JC-1 dye staining showed that mitochondrial membrane potential in DP2-EC and DP3-ECs was unchanged after treated with 600 ng/mL Tb4 (Fig. 5C, D).

### Tb4 (600 ng/mL) protects dia-hiPSC-EC from hypoxic damage

**Fig. 6:**
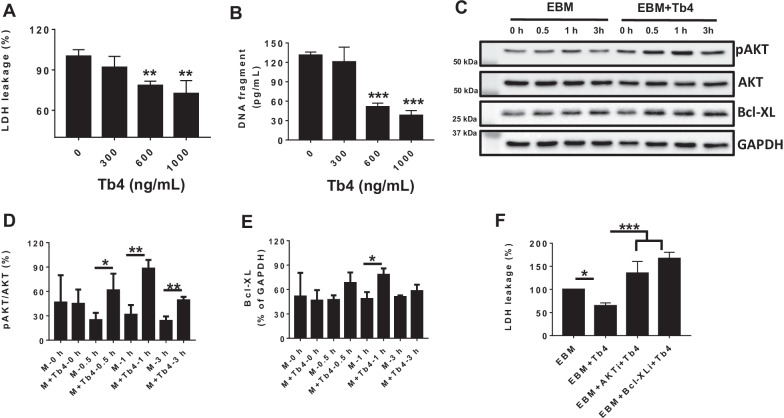
Tb4 improves dia-hiPSC-ECs viability against hypoxic injury in vitro. **A** The concentrations of lactate dehydrogenase (LDH) in the cell culture medium were measured in the absence or presence of Tb4 and presented as a percentage of the measurements obtained in the absence of Tb4. **B** The concentration of DNA fragments in the cell culture medium were measured in the absence or presence of 600 ng/mL Tb4. **C** Representative Western Blot images of dia-hiPSC-ECs cultured in EBM supplemented with or without 600 ng/mL Tb4 for protein expressions of pAKT, AKT, and Bcl-XL. Protein expression level of GAPDH was used as an internal control. Cells were cultured under hypoxic condition and were harvested at 0, 0.5, 1, and 3 h after treatment. **D** Quantification of pAKT protein expressions, which were expressed as percentages of AKT protein levels after normalized with GAPDH protein. **E** Quantification of Bcl-XL protein expressions, which were presented as percentages of GAPDH protein levels. **F** Pre-treatment with AKT inhibitor (AKTi) or Bcl-XL inhibitor (Bcl-XLi) blocked cytoprotective effect of Tb4. (Values are presented as the means ± SD. (**A**, **B**, **F**: *n* = 4, One-Way ANOVA; D&E: *n* = 3 for each sample and time point, independent *T*-test. *p < 0.05; **p < 0.01; ***p < 0.001)

To determine cyto-protection effect of Tb4 on ECs, dia-hiPSC-ECs were cultured in EBM supplemented with or without Tb4 in hypoxic condition for 24 h. Treatment with Tb4 (600 and 1000 ng/ mL) significantly reduced LDH leakage (Fig. 6A) and DNA damage (Fig. 6B). Notably, Tb4 significantly upregulated both AKT activity and Bcl-XL levels under hypoxic condition up to 3 h in vitro (Fig. 6C–E). Furthermore, either AKT or Bcl-XL inhibitor totally blocked cytoprotective effect of Tb4 against hypoxia injury (Fig. 6F). Thus, Tb4 appeared to protect hiPSC-CMs from hypoxia-induced cellular damage by upregulating AKT activity and Bcl-XL protein expression.

### Tb4 (600 ng/mL) treated Dia-hiPSC-EC improves angiogenic potential for treatment of HLI

**Fig. 7:**
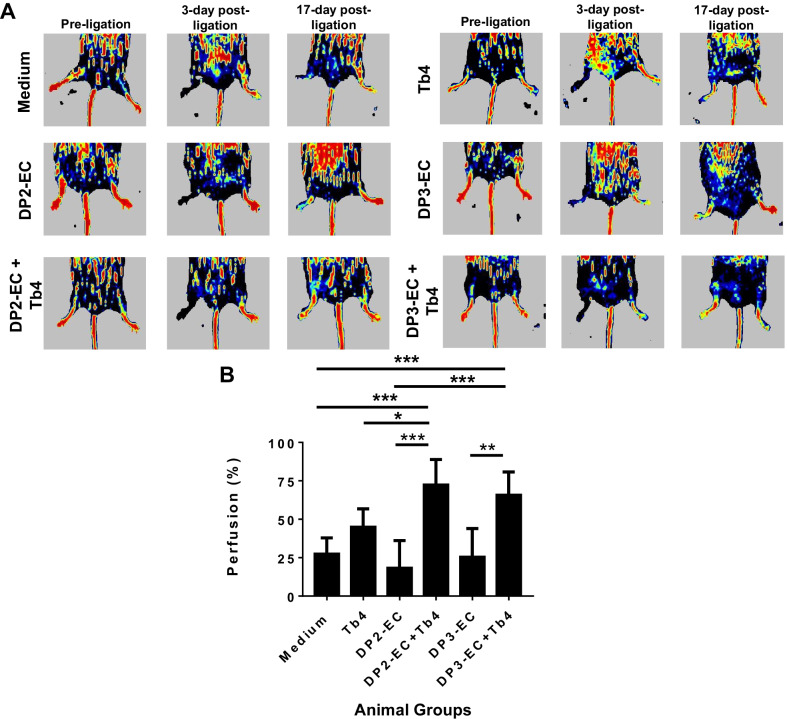
Tb4 improves reparative potency of dia-hiPSC-ECs for treatment of ischemic limb disease in mice with T2DM. **A** Laser Doppler imaging of mouse limbs before femoral artery ligation, 3 days (i.e., at the time of treatment administration), and 17 days (i.e., at the 14 days medium or cell injection) after femoral artery ligation. **B** Recovery of right limb perfusion was expressed as a percentage of measurements in the uninjured contralateral limb. Values are presented as means ± SD. One-way ANOVA (*p < 0.05; **p < 0.01; ***p < 0.001)

The potential of Tb4 treated and non-treated dia-hiPSC-ECs for treatment of ischemic disease was evaluated in a mouse model of HLI (Fig. 7A) [25]. Perfusion was 27.5 ± 10.4% in the control group, 18.3 ± 17.8% in the DP2-EC + microsphere group, and 25.5 ± 18.4% in the DP3-EC + microsphere group, which were significantly lower than those of the DP2-EC + Tb4-microsphere group (72.5 ± 16.4%, *p* < 0.001 vs medium, DP2-EC, and DP3-EC groups) and DP3-EC + Tb4-microsphere group (65.8 ± 15.0%, *p* < 0.01 vs medium, DP2-EC, and DP3-EC groups) (Fig. 7B). Although Tb4-microsphere group increased perfusion to 45 ± 11.8%, no significant improvement was achieved as compared with the Medium, DP2-EC, and DP3-EC groups and was significantly lower than the DP2-EC + Tb4-microsphere group (*p* < 0.05).

**Fig. 8:**
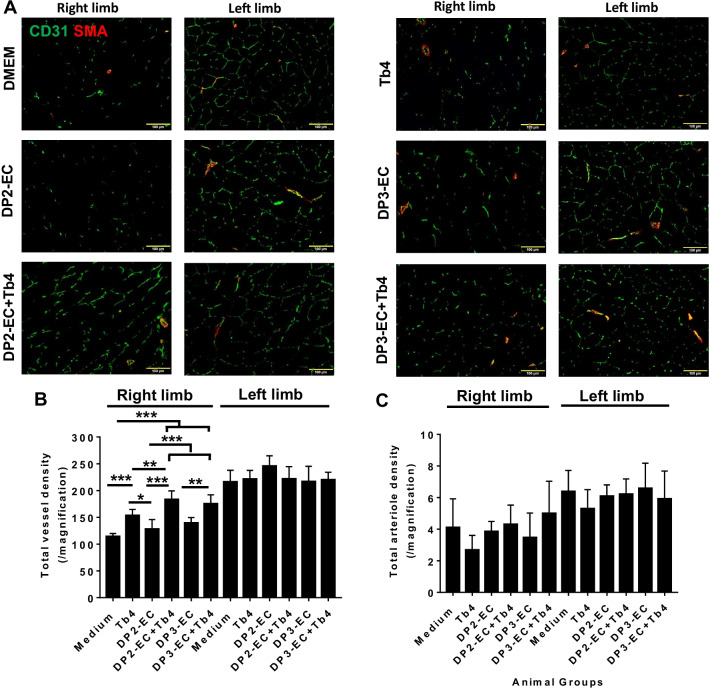
Quantification of vascular density in mouse hind limbs. **A** Fluorescence staining for CD31 and smooth muscle actin (SMA) in the ischemic limb (right leg) and uninjured limb (left leg) of animals treated with basal medium or hiPSC-ECs after femoral artery ligation. (Bar = 100 μm). **B** Vessel density and **C** arteriole density in ischemic limbs (right limb) and uninjured contralateral limbs (left limb) (*n* = 6 for each animal group). Values are presented as means ± SD. One-way ANOVA (**p < 0.01; ***p < 0.001)

Assessments in cryo-sections stained for CD31 (detecting both hiPSC-EC and mouse EC) and SMA (Fig. 8A) indicated that total vessel density was significantly higher in the ischemic limbs of animals in the DP2-EC + Tb4-microspheres (183.4 ± 15.9) and DP3-EC + Tb4-microspheres groups (175.3 ± 16.8) than the medium (114.2 ± 5.7, *p* < 0.001) or or DP2-EC + microspheres (128.1 ± 17.9, *p* < 0.001) or DP3-EC + microspheres (139.5 ± 10.1; *p* < 0.001 or *p* < 0.01) group (Fig. 8B). Although total vessel density in Tb4 group (153.6 ± 11.1) was significantly higher than the medium (*p* < 0.001) or the DP2-EC group (*p* < 0.05), it was significantly lower than the DP2-EC + Tb4 group (*p* < 0.01). Arteriole densities in the ischemic limbs of animals were similar among all 5 groups (Fig. 8A, C). Collectively, these observations suggest that Tb4 treated dia-hiPSC-ECs had better angiogenic potential in restoring perfusion and stimulating neovascularization in ischemic limb of mouse with diabetes.

**Fig. 9:**
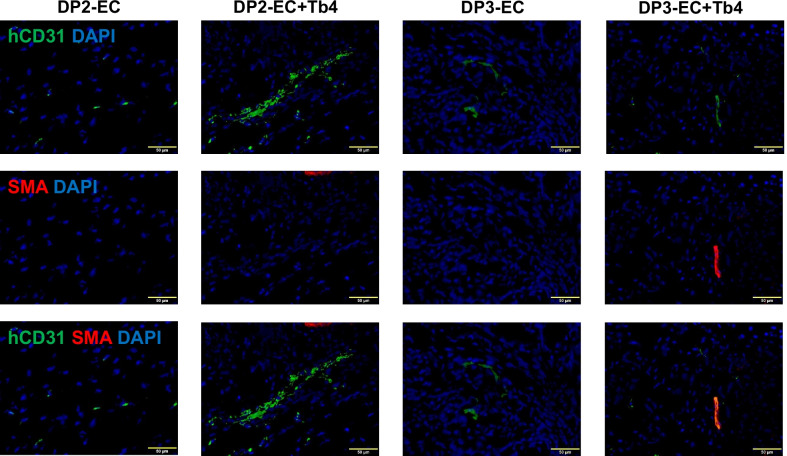
Fluorescence staining for human-specific CD31 (hCD31) and SMA expression in the injured limbs of hiPSC-EC treated animals (Bar = 50 µm)

Tissue sections stained for the specific hCD31 and SMA indicated that the transplanted dia-hiPSC-ECs formed capillaries in all animal groups that received dia-hiPSC-EC transplantation and some integrated into arterioles (Fig. 9). These data suggest that transplanted hiPSC-ECs can contribute to capillary and arteriole formation in ischemic limbs of KK mouse.

## Discussion

Our previous studies showed that DP2-ECs and DP3-ECs, which were derived from patients with T2DM, had poor cell proliferative capability, increased senescence, and impaired mitochondrial function and angiogenic potential [12]. These signature phenotypes suggest that DP2-ECs and DP3-ECs can be used as disease models for studying endothelial dysfunction in diabetes. In the current study, we found that Tb4 enhanced dia-hiPSC-EC viability and proliferation, inhibited senescence, and improved reparative potency of dia-hiPSC-ECs for treatment of ischemic limb disease in mice with T2DM.

Studies have shown that Tb4 activates the survival kinase AKT through integrin-linked kinase (ILK) [16]. AKT is a key regulator of EC survival, proliferation, metabolism, and angiogenesis [35, 36], while Vascular endothelial growth factor-165 is a potent mitotic factor on ECs through PI3K/AKT pathway, which is important in mediating EC proliferation, viability, senescence and angiogenesis [37]. Thus, convergences of VEGF and Tb4 in activating AKT and associated signaling pathways may be responsible for increased dia-hiPSC-EC proliferation, reduced EC senescence, enhanced EC survival under hypoxia, and improved reparative potency of dia-hiPSC-ECs.

Activated AKT can subsequently upregulates transcription factors that increase the expression of caspase inhibitors, including Bcl-XL, to promote cell survival. These results are consistent with our previous studies which showed that Tb4 enhanced cardiomyocytes (CMs) and mesenchymal stem cell (SMCs) viability and survival under hypoxia in vitro and infarcted myocardial in vivo [18, 19].

The effect of Tb4 on dia-hiPSC-ECs is dose-dependent and a higher Tb4 dosage (600 ng/mL) is required. This is consistent with 2 studies showed that 600 or 1000 ng/mL Tb4 is needed to exert cyto-protection and increases proliferation on CMs or SMCs [18, 19]. Surprisingly, in vitro, a low dose Tb4 (10 ng/mL) was shown to increase migration and angiogenic factor secretion of primary endothelial progenitor cell (EPC) isolated from Zucker diabetic fatty rats [24]. However, transplantation of this low dose Tb4 treated EPCs failed to improve left ventricular pump function in diabetic rats after MI. This study suggests that a higher dosage of Tb4 may be needed to improve therapeutic potential of diabetic ECs for treatment of ischemic diseases, such as ischemic heart disease or ischemic limb disease, especially in diabetes.

In addition, the current study also showed that Tb4 reduced endothelin-1 and MMP-1 production in dia-hiPSc-ECs. Endothelin-1 is a potent vasoconstrictor and has been shown to be involved in the development of atherosclerosis [38], hypertension[39], microvascular dysfunction in diabetes [40], stroke etc. [41]. It is unkown through which mechanism that Tb4 reduces endothelin-1 production secreted by dia-hiPSC-ECs and may need further exploration. Inhibition of AKT has been shown to up-regulate MMP-1 expression in human dermal fibroblasts [42]. Our study shows that enhanced AKT activity stimulated by Tb4 down-regulates MMP-1 production. This supports senescence study which shows that Tb4 reduces senescence in dia-hiPSC-ECs, as MMP-1 is a marker that makes up the SASP.

It seems that Tb4 has no effect on mitochondrial membrane potential, intercellular adhesion molecule expression, and glycine homeostasis, as JC-1 dye intensity, ICAM-1 and mSHMT protein expression levels were unchanged after Tb4 treatment. Our prior study showed that mSHMT, not cytoplasmic serine hydroxymethyltransferase (cSHMT) and glycine transporter protein expression, significantly reduced in dia-hiPSC-ECs, suggesting that mSHMT may be responsible for significantly reduced intracellular glycine concentration and dysregulated glycine homeostasis in dia-hiPSC-ECs [12].

The current and previous studies both showed that diabetic ECs have poor reparative potency for treatment of ischemic limb diseases in either NOD-SCID or diabetic mice. This implies that autologous ECs differentiated from hiPSCs, which are derived from patients with diabetes, are not suitable cell type for treatment of ischemic diseases in patients with diabetes. To achieve therapeutic effect, treatments to improve endothelial function, cell viability, and angiogenesis shall be performed. Tb4 may offer an easy and cost-effect way to improve diabetic EC viability and proliferation, inhibit senescence, and improve reparative potency.

Tb4 is known to have other beneficial effects in diabetes mouse models. It can improve glucose intolerance, reduce insulin resistance [43] and ameliorate hyperglycemia induced renal damage [44]. Our study has now demonstrated a possible useful effect in peripheral vascular disease and lower- limb ulcers. One limitation of the current study is that it is unknown through which mechanism that Tb4 reduces endothelin-1 production by dia-hiPSC-ECs and may need further exploration.

## Conclusions

600 ng/mL Tb4 improves endothelial dysfunction through enhancing dia-hiPSC-EC viability and proliferation, reducing senescence and endothelin-1 and MMP-1 secretion, and improving reparative potency of dia-hiPSC-ECs for treatment of ischemic limb disease in mice with T2DM. These data support to use Tb4 as a potential drug for treatment of systemic endothelial dysfunction in diabetes with peripheral vascular disease.

## Data Availability

The data supporting the conclusions of this article is included within the article.
